# Robotic Off-Clamp Simple Enucleation Single-Layer Renorrhaphy Partial Nephrectomy (ROSS): Surgical Insights after an Initial Experience

**DOI:** 10.3390/jcm12010198

**Published:** 2022-12-27

**Authors:** Riccardo Bertolo, Chiara Cipriani, Matteo Vittori, Riccardo Campi, Juan Garisto, Michele Di Dio, Filippo Annino, Pierluigi Bove

**Affiliations:** 1 Department of Urology, San Carlo di Nancy Hospital, 00165 Rome, Italy; 2 Unit of Urological Robotic Surgery and Renal Transplantation, Careggi Hospital, University of Florence, 50134 Florence, Italy; 3 Department of Experimental and Clinical Medicine, University of Florence, 50134 Florence, Italy; 4 Division of Urology, Veteran Affairs Boston Healthcare System, Boston, MA 02130, USA; 5 Division of Urology, Brigham and Women’s Hospital, Boston, MA 02115, USA; 6 Department of Urology, SS Annunziata Hospital, 87100 Cosenza, Italy; 7 Urology Unit, Azienda USL Toscana Sud-Est, San Donato Hospital, 52100 Arezzo, Italy; 8 Urology Unit, Department of Surgery, Tor Vergata University of Rome, 00133 Rome, Italy

**Keywords:** partial nephrectomy, enucleation, clampless, off-clamp, technique, surgical experience

## Abstract

Robotic technology allows the beginner surgeon to approach minimally-invasive partial nephrectomy (PN) avoiding the otherwise long learning curve of pure laparoscopy. The present video-article reported the surgical technique and the outcomes of the first 11 cases performed by a young surgeon starting with the experience of robotic PN. Transperitoneal robotic PN, with an off-clamp approach, a simple enucleation technique, and a single-layer medullar renorrhaphy was performed uneventfully in all cases but one, with comparable outcomes to the available literature. With the present experience, we are trying to give the reader a different point of view of the current knowledge. In our series, off-clamp robotic PN was not chosen while looking for a better functional outcome, but rather as a “forced” choice within the specific “in training” setting the interventions were performed in. We underline how the off-clamp approach was the way to cut out the potential for vascular complications derived from the application/removal of the clamp itself on the renal artery. Indeed, when Scanlan bulldogs are not available, one of the limits of robotic PN is that the first surgeon is not autonomous in placing/removing the clamp. We found that tumour enucleation resection technique had the perfect synergistic effect in maximizing the perioperative vision, and thus the safety, notwithstanding the clampless approach.

## 1. Introduction

The guidelines recommend partial nephrectomy (PN) as the gold-standard treatment for patients with localized T1 renal tumours, so that the technique used to excise the tumour has paramount importance to achieve the goals of perioperative safety, oncological efficacy, and maximized functional preservation.

With the diffusion of technology, robot-assisted PN (RAPN) has progressively become the preferred approach both in experts’ and naïve surgeons’ hands.

The advantages of a robotic approach have been described for both the categories of expertise: namely, robotics expands the indications to nephron-sparing surgery for highly complex renal masses in expert hands, while simply allows the beginner to perform minimally-invasive PN avoiding the otherwise long learning curve of pure laparoscopy. The literature is mostly focused on RAPN series performed by expert surgeons [1].

In recent years, robotic technology has been allowing the beginner surgeon to approach minimally-invasive PN, avoiding the otherwise long learning curve of pure laparoscopy. The aim of the present video-article was to report the surgical technique and the outcomes of the first cases performed by a young surgeon starting with the experience with robotic PN, specifically focusing on tips and tricks aimed to maximize the safety of the procedure.

## 2. Materials and Methods

We herein report the surgical technique and the outcomes of the first cases performed by a beginner surgeon. Preoperative data including age, sex, body mass index, hemoglobin, Charlson’s Comorbidity Index, serum creatinine and eGFR, operated kidney % contribution to renal function (assessed by radionuclide renal scan), tumour size and complexity (assessed by RENAL score) were collected. Cases were consecutive, but in case of high complexity as assessed by RENAL score (>9), the procedure was performed by the chief surgeon. Patients had baseline estimated glomerular filtration rate > 60 mL/min (stage I/II chronic kidney disease). Intraoperative and postoperative data including operative time, intra-abdominal pressure, use of valveless trocar, eventual clamping on demand (ischemia time in case of clamping), resection technique (enucleation vs. enucleo-resection vs. resection), renorrhaphy time and technique, use of hemostatic agents, estimated blood loss, use of drainage and occurrence of intraoperative adverse events were analyzed.

## 3. Results

Eleven patients were included in the present analysis. Three complex cases (RENAL score > 9) within the time span considered were operated by the chief surgeon. Median preoperative tumour size was 2.8 cm (IQR 1.9–4.0). Median tumour complexity as assessed by the RENAL nephrometry score was 7 (5–8). Complete patients’ and tumours’ characteristics are reported in Table 1. Transperitoneal RAPN, with an off-clamp approach (but including main renal artery dissection), a simple enucleation technique, and a single-layer medullar renorrhaphy was performed in all cases (ROSS). The ROSS surgical technique is detailed in the accompanying video.

All the cases but one were conducted according to a pure enucleation strategy. Namely, one tumour rupture occurred that was angiomyolipoma at intraoperative frozen section. This was the case of a lesion with no robust pseudo-capsule, that prevented an uneventful tumour enucleation technique. Nevertheless, macroscopic R0 surgery was achieved at the end of the resection and final pathology confirmed the benign histology. Clamping on demand of the renal artery was never required but we remark that the surgeon always dissected the renal artery. Single-layer medullar renorrhaphy was performed in all cases. No drain was placed at the end. Haemostatic agents were used according to surgeon’s preference.

No clinically-relevant postoperative complications occurred (two patients had fever, one requiring non-routine-use of an intravenous antibiotic). Perioperative and postoperative outcomes are detailed in Table 2 and Supplementary Table S1.

## 4. Discussion

The debate over the merits and limitations of different techniques during RAPN has been reinforced by recent studies. An “old school” PN would have included (1) renal artery clamping as a standard step before tumour resection; (2) excision of a margin of peritumoral tissue to ensure negative margins; (3) a double-layer renorrhaphy, focused on minimizing the occurrence of complications [2].

Such dogmas have been revolutionized by the literature of the last decades. In particular, (1) with the goal of reducing renal damage, off-clamp technique during RAPN has been described to excise the tumour without clamping the renal artery [3]; (2) as the amount of functional parenchymal mass preserved during PN has been shown to be one of the strongest modifiable predictors of functional recovery after surgery [4], some authors argued that tumour enucleation may have distinct benefits over a “traditional” PN without compromising the oncologic safety [4]; (3) the renorrhaphy techniques have evolved over time toward the concept of a “nephron-sparing renal reconstruction”: beyond closing the renal defect, the goal is again to maximize the vascularized parenchyma [2]. Published literature provides data that give us indications about the respective “ideal” technique: (1) recently completed randomized trials would discourage the continued use of off-clamp RAPN, given the absence of clearly superior functional outcomes [5]; (2) prospective multicentre studies showed how resection techniques impact on perioperative and early functional and oncologic outcomes in patients with localized renal masses [6]; (3) pooled literature analysis showed a functional advantage from performing a single-layer renorrhaphy, without increasing the likelihood of complications after PN [2].

With the present experience, we are trying to give the reader a different point of view of the current knowledge. In our series, off-clamp RAPN was not chosen while looking for a better functional outcome, but rather as a “forced” choice within the specific “in training” setting in which the interventions were performed.

We believe that the unavailability of Scanlan bulldogs (Health Aid Company, Inc., Tampa, FL, USA) makes the application (and removal) of the clamp one of the riskier steps of the procedure.

Therefore, an off-clamp approach was chosen. Paradoxically, this occurred even at the start of the experience. As such, it has been a common trend that only after the learning curve is overcome does the surgeon does embark on no ischemia techniques.

Rather, in our experience, off-clamp was the way to cut out the potential for vascular complications derived from the application/removal of the clamp itself. When Scanlan bulldogs are not available, one of the limits of robotic PN is that the first surgeon is not autonomous in placing/removing the clamp.

As concerning the resection technique, tumour enucleation had the perfect synergistic effect in maximizing the perioperative vision, and thus the safety.

The video shows how the vessels emerging from the resection bed could bleed but how they were managed simply by bipolar coagulation while following the enucleation plane.

Notwithstanding an off-clamp approach, enucleation technique allowed to excise the tumour with optimal visualization of its contours. Beyond being synergic with the clampless technique, it importantly sponsored a “nephron-sparing”, selective renorrhaphy [2]. Indeed, a nephron-sparing bluntly developed tumour excision, as in the case of tumor enucleation (or minimal-margin PN), facilitates an anatomical nephron-sparing renal reconstruction. Virtually, no healthy tissue is removed. If some experts remain sceptical about the real advantages of tumour enucleation, arguing that it might lead to insignificant differences in postoperative renal function and complications as compared to standard PN, at the cost of a higher risk of tumour violation, we underline with our experience how such a technique can be the way for a beginner to safely and effectively perform RAPN, even with an off-clamp approach.

Interestingly, although the very initial experience, tumor excision was conducted in an anatomical way, paving the surgeon’s intent, what we refer to as “resection strategy”.

Supporting our preliminary experience, a prospective multicentric study has shown how resection techniques do impact on perioperative (and early functional and oncologic) outcomes in patients with localized renal masses. Minervini et al. introduced the standardized reporting of resection techniques during PN (the Surface–Intermediate–Base (SIB) score) [7] and found the resection technique as the only significant predictor of positive surgical margins and one of the strongest predictors of ≥grade 2 surgical complications and trifecta achievement [6].

## 5. Conclusions

In conclusion, our experience shows how robotics can be a facilitator of surgery. While knowing relatively basic surgical principles, PN for intermediate/low complexity renal masses can be performed in a safe and effective way from the start of the experience. Specifically, in our setting, off-clamp approach was felt to be the best compromise to maximize safety. On the other hand, we acknowledge that surgeon’s preference has a role. Moreover, there is no functional advantage from going off-clamp based on randomized trials (RCTs) but off-clamp remains an option supported by the fact that the same RCTs did not reveal higher likelihood of complications related to the approach [5].

## Figures and Tables

**Table 1:** Distribution of baseline characteristics of patients.

	ROSS (No. Cases = 11)
**Patient’s features**	
Age, years	64 (55–70)
No. of Males	6 (54.5)
BMI, kg/m^2^	26 (24–29)
Hemoglobin, g/dL	14.3 (13.3–15.2)
Charlson’s Comorbidity Index	0 (0–1)
Serum creatinine, mg/dL	0.8 (0.7–1.0)
eGFR, mL/min	87 (76–96)
Operated kidney contribution to renal function, %	48 (46–51)
**Tumor’s features**	
Diameter, cm	2.8 (1.9–4.0)
R.E.N.A.L. score	7 (5–8)

Categorical variables were summarized as absolute and relative frequencies, while numerical variables as median and interquartile range (IQR). IQR and percentages were reported in brackets. ROSS: Robotic off-clamp simple enucleation single-layer renorrhaphy partial nephrectomy; BMI: body mass index; eGFR: estimated glomerular filtration rate.

**Table 2:** Distribution of intraoperative outcomes.

	ROSS (No. Cases = 11)
Operative time, min	140 (110–170)
Intra-abdominal pressure, mmHg	12 (12–12)
No. Use of Airseal^®^	11 (100)
No. Clamping on demand	0 (0)
Ischemia time, min	0 (0)
SIB score	
No. Enucleation	8 (72.7)
No. Enucleo-resection	2 (18.2)
No. Resection	1 (9.1)
Suture time, min	10 (8–13)
Renorrhaphy technique	
No. Sutureless	0 (0)
No. Single-layer medullar only	10 (90.9)
No. Single-layer cortical only	1 (9.1)
No. Double-layer (medullar and cortical)	0 (0)
No. Haemostatic agents	6 (54.5)
Estimated blood loss, mL	120 (100–200)
No. Drain	0 (0)
Intraoperative adverse events *	(0)

Categorical variables were summarized as absolute and relative frequencies, while numerical variables as median and interquartile range (IQR). IQR and percentages were reported in brackets. ROSS: robotic off-clamp simple enucleation single-layer renorrhaphy partial nephrectomy; SIB: surface–intermediate–base score. * As classified by the ICARUS Classification System Working Group. Assessing, grading, and reporting intraoperative adverse events during and after surgery. Br J Surg. 2022 Mar 15;109(4):301–302. doi: 10.1093/bjs/znab438.

## Data Availability

The datasets generated during and/or analyzed during the current study are available from the corresponding author on reasonable request.

## Supplementary Materials

The following supporting information can be downloaded at: https://www.mdpi.com/article/10.3390/jcm12010198/s1, Table S1. Distribution of post-operative and final pathology variables.
