# Supplementary material for: Robotic Off-Clamp Simple Enucleation Single-Layer Renorrhaphy Partial Nephrectomy (ROSS): Surgical Insights after an Initial Experience
Source: J Clin Med. 2022 Dec 27;12(1):198. doi: 10.3390/jcm12010198 (PMC9821137; doi:10.3390/jcm12010198)
Supplement: Supplementary file 1 [file jcm-12-00198-s001.zip › jcm-2069712-supplementary.pdf]

Table S1. Distribution of post-operative and final pathology variables.

	ROSS (no. cases = 11)
**Post-operative variables**	
No. Clavien–Dindo ≥ 2 complications	1 (9.1)
Clavien–Dindo complication grade	
1	1 (9.1)
2	1 (9.1)
3 - 5	0 (0)
No. Embolization	0 (0)
No. Reintervention	0 (0)
Length of stay, days	3 (3 - 4)
1st POD Hemoglobin, g/dl	12.6 (11.5 - 13.8)
3rd POD Hemoglobin, g/dl	12.5 (11.4 - 13.0)
Max Postoperative Hemoglobin drop, g/dl	-1.8 (-2.2 - -1.0)
3rd POD Δ eGFR, ml/min	-4.5 (-2.2; +15.0)
3rd POD Δ eGFR, %	-6.5 (-14.7; +2.9)
No. 3rd POD Δ eGFR > 25%	0 (0)
**Final pathology variables**	
Tumor size, cm	2.7 (1.8 - 4.2)
No. Malignant Histology	8 (72.7)
Tumor Stage (only malignant lesions)	
No. T1a	6 (75.0)
No. T1b	1 (12.5)
No. T2	0 (0)
No. T3a	1 (12.5)
No. ISUP grade 3-4 (only malignant lesions)	2 (25.0)
No. Positive Surgical Margins	0 (0)*

Categorical variables were summarized as absolute and relative frequencies, while numerical variables as median and interquartile range (IQR). IQR and percentages were reported in brackets. ROSS: robotic off-clamp simple enucleation single-layer renorrhaphy partial nephrectomy; POD: post-operative day; eGFR: estimated glomerular filtration rate; ISUP: International Society of Urological Pathology. *one tumour rupture occurred that was angiomyolipoma at intraoperative frozen section. Macroscopic R0 surgery was achieved at the end of the resection. Final pathology confirmed the benign histology, but Rx margin status was assigned.
